# Diverting organic waste from landfills via insect biomanufacturing using engineered black soldier flies (*Hermetia illucens*)

**DOI:** 10.1038/s42003-024-06516-8

**Published:** 2024-07-24

**Authors:** Kate Tepper, Owain Edwards, Anwar Sunna, Ian T. Paulsen, Maciej Maselko

**Affiliations:** 1 Applied BioSciences, Macquarie University, Sydney, NSW Australia; 2 ARC Centre of Excellence in Synthetic Biology, Macquarie University, Sydney, NSW Australia; 3 EntoZyme PTY LTD, Sydney, NSW Australia; 4 School of Natural Sciences, Mascquarie University, Sydney, NSW Australia; 5 Biomolecular Discovery Research Centre, Macquarie University, Sydney, NSW Australia

**Keywords:** Genetic engineering, Environmental biotechnology

## Abstract

A major roadblock towards the realisation of a circular economy are the lack of high-value products that can be generated from waste. Black soldier flies (BSF; *Hermetia illucens*) are gaining traction for their ability to rapidly consume large quantities of organic wastes. However, these are primarily used to produce a small variety of products, such as animal feed ingredients and fertiliser. Using synthetic biology, BSF could be developed into a novel sustainable biomanufacturing platform to valorise a broader variety of organic waste feedstocks into enhanced animal feeds, a large variety of high-value biomolecules including industrial enzymes and lipids, and improved fertiliser.

## Abstract (web summary)

This perspective describes how insects, such as Hermetia illucens, which are already used to process organic wastes at industrial scales, could be developed as a synthetic biology platform for sustainable biomanufacturing and bioremediation.

## Introduction

Management of organic waste is a major global challenge. Currently, ~40–70% of organic waste is disposed of in landfills^1–4^, where it is tightly compacted to economise space. This leads to the anaerobic microbial decomposition of organic waste into methane, a greenhouse gas (GHG) that is 28 times more potent than carbon dioxide^5^. Consequently, the solid waste sector is estimated to be responsible for 5% of global annual CO_2_-equivalent emissions, with the majority of this due to the methane generated from landfilling organic waste^6^. Continued rising GHG emissions have led to the Intergovernmental Panel on Climate Change reporting that we are facing an imminent climate disaster as we expect to surpass the 1.5 °C critical global warming threshold in 2031^7^. Urgent intervention from policy makers, bolstered by innovative technologies to handle organic waste, is imperative to achieve net-zero annual GHG emissions.

Adding to the problem are municipal biosolids, another significant source of organic waste. Municipal biosolids are nutrient-rich organic by-products from treating sewage at municipal wastewater treatment plants. Despite their high nutrient content and utility as an alternative to synthetic fertiliser, there is an increasing body of research demonstrating that biosolids are contaminated with hazardous chemicals^8–10^. Notable examples include heavy metals, microplastics, per- and poly-fluoroalkylated substances (PFAS), pharmaceuticals and 35 chemicals on the EPA priority pollutant list^10,11^. Regulations are increasingly restricting using biosolids as fertiliser^12^, and alternative management practices are required for their disposal.

Challenges to sustainable waste management are particularly severe in developing countries where public services for waste management can be limited or non-existent. Organic waste is often openly dumped, which contributes to the proliferation of pathogens and pests, contaminates water used for drinking and irrigation and results in habitat destruction^6^. Additionally, a large portion of biomass from food crops is non-edible and is frequently managed by open burning that results in unsafe air quality^6,13^.

Many developed nations are implementing organic waste landfill bans to hasten the adoption of waste management strategies that align with the public good. Yet scalable and cost-effective strategies are lacking as the products that can be generated from waste, such as compost, biofuels and animal feed have limited value and there are significant upfront and maintenance costs required to develop the infrastructure to collect, sort, and fully utilise organic waste.

The circular economy is an economic system that aims to minimise waste and maximise resource efficiency by promoting the reuse, recycling and regeneration of materials in continuous cycles, and offers a promising framework to address these challenges. Technological innovations that increase the variety and value of products that can be regenerated from organic wastes will incentivise greater organic waste circularisation and provide an opportunity for entrepreneurs to valorise organic waste.

Synthetic biology has the potential to provide many of the needed technological innovations that could address climate change and sustainability^14^. Though underdeveloped in comparison to microbial synthetic biology, insect synthetic biology could be used to clean up and valorise organic waste by engineering insects that are already adept at processing organic wastes as a platform for biomanufacturing and bioremediation. This type of insect biomanufacturing could convert organic wastes into higher value products such as enhanced feed, industrial biomolecules and high-quality fertiliser. Globally, it is estimated that the animal feed market is worth USD $500 billion^15^. The global industrial enzyme and lipid market is worth USD $6.95 billion^16^ and USD $13.63 billion^17^, respectively. The global fertiliser market is worth USD $207 billion^18^.

In this perspective we describe why insects are a very promising synthetic biology platform to improve sustainable biomanufacturing. We describe the opportunity, advantages and disadvantages and how to mature insect biomanufacturing with a focus on black soldier flies.

## Modern biomanufacturing

Biomanufacturing is the use of biological systems and their cellular machinery to generate valuable biomolecules with utility in industries such as agriculture, food, pharmaceutical, material and energy^19^. Typically, mono-cultures of microbes, fungi and cell lines are used for commercial biomanufacturing, predominantly via large scale bioreactor-based fermentation. To a lesser extent, plant and algal systems are also used for biomanufacturing. Products include small molecule primary metabolites such as acetone, butanol, ethanol, amino acids, and organic acids^20^; and complex secondary metabolites such as antibiotics, flavourings, colourings and fragrances^21–23^. Genetically engineered organisms are used to produce many larger high-value proteins including insulin^24^, growth hormones^25^, erythropoietin^26^, industrial enzymes^27^, enzymes for molecular biology^19^, monoclonal antibodies^28^ and vaccines^29^. Microbial fermentation systems and cell lines have been particularly invaluable to commercial biomanufacturing due to the relatively simple rearing conditions, fast growth rates, ease of storage, established genetic manipulation methodologies and diverse metabolic potentials.

The United Nation’s Sustainable Development Goals (SDGs)^30^ have catalysed a shift in focus to develop technologies that reduce emissions and pollution, and to replace processes based on fossil fuels with sustainable bio-based approaches. This includes changing approaches to the production of high-value biomolecules to utilise waste as feedstocks. Current industrial biomanufacturing strains are resource and energy intensive as they typically require sophisticated infrastructure, large quantities of water, feedstocks predominantly sourced from food crops and feedstock preparation that is resource and energy intensive^31^. Plant molecular farming is less energy and resource intensive than bioreactor-based biomanufacturing as it can directly fix carbon dioxide to produce valuable biomolecules, and could be used as a carbon capture technology for emissions reductions^32^. However, it has a large land-use footprint and requires large quantities of water and fertiliser that could otherwise be used for growing food. It could impact food security as well as deforestation and biodiversity as more land is cleared to grow these plants. Furthermore, plants grow more efficiently in the field and to commercialise any one transgenic plant for growth outside of physically contained greenhouses requires long and expensive regulatory hurdles^33^. Effective transgene biocontainment strategies are limited and transgenic plants growing as volunteer crops have contaminated food supply chains; leading to expensive recalls, food wastage and damages to public trust^34,35^. Diversification of biomanufacturing technologies for greater sustainability is therefore required. The UN’s SDGs will be described in more detail in the ‘Addressing the SDGs’ section.

## Insect biomanufacturing

Beekeeping for honey, beeswax and pollination has existed since the dawn of agriculture, with evidence of humans using beeswax found across Neolithic Europe, the Near East, and North Africa^36^. Silk production from the cocoon of the silkworm (*Bombyx mori*) originated in China in the 3rd millennium BC and is a high-value product to this day. Recently silkworms have been used as a platform for synthetic biology and nanotechnology to generate products that harness the properties of silk fibre. It has been used to generate high-value protein therapeutics, spider silk, antimicrobial bandages, drug delivery systems, dissolvable and biocompatible medical implants, scaffolding for tissue engineering, precision agriculture for the controlled delivery of bioactives to plants, among other applications^37–42^. Extracts from the Coccoidea superfamily of scale insects are used for colourful dyes and resins^43^. Products include kermes, Polish cochineal, Mexican cochineal, lac dye and shellac. These have important historical, cultural and economic value as the low production output and low supply of these eye-catching products were sought after by nobility.

Insect cell lines and insect larvae/pupae are often used in molecular biology research and commercial biomanufacturing to transiently overexpress proteins of interest^44^. They are less likely to become contaminated by human pathogens than mammalian cell lines and they provide many of the post-translational modifications required for eukaryotic protein stability and function. Approximately 1% of FDA/EMA approved protein therapeutics are biomanufactured by insect cell lines^44^. Examples include: vaccines for COVID-19 (Novavax)^45^, influenza (Flublok)^46^ and cervical cancer (Ceravix)^47^; cellular immunotherapies for prostate cancer (Provenge)^48,49^; and adenovirus production for familial lipoprotein lipase deficiency gene therapy (Glybera)^50^. Cocoon Biosciences, a spin out company of Algenex, is using cabbage looper (*Trichoplusia ni*) pupae biomanufacturing process to generate growth factors for the cultivated meat industry and enzymes for industrial biotechnology^51^.

Insects as a source of food is a delicacy in many cuisines across the world and will likely benefit from economies of scale to make protein that is more affordable than more conventional livestock^52^. Though insects are not regarded as a delicacy in the West, they are gaining interest as a sustainable source of protein due to their high feed conversion efficiency of pre-consumer food wastes^53^. Insects are commonly utilised as nutritious feed for pets and livestock^54,55^. Insect meal is set to become less expensive than other protein feed products such as fishmeal^56,57^. Though it is still more expensive than soy protein, it is considered a more sustainable option^58^.

Various industrial biomanufacturing applications of cell line and microbial systems and whole insects are shown in Tables 1 and 2, respectively.

**Table 1:** Examples of cell line and microbial culture systems used for commercial biomanufacturing

Organism	Substrate	Product examples	Commercial examples
Bacteria: *Escherichia coli, Bacillus subtilis, Pseudomonas putida, Streptomyces spp*.	Carbon: starch and cellulosic biomass Nitrogen: ammonium/nitrate salts, urea, soy protein, amino acids, yeast extract Mineral and trace elements	Primary metabolites: acetone, butanol, ethanol, amino acids and organic acids	DuPont (US) Gevo (US) Lanzatech (NZ/US)
		Protein therapeutics: Insulin, growth hormone	Genentech (US) Eli Lilly (US)
		Enzymes: Amylase, lipase, protease and other industrial enzymes. Enzymes for molecular research	Novozyme (Denmark) DuPont (US) New England Biolabs (US)
		Bt (*Bacillus thuringiensis*) insecticide	Bayer (Germany)
		Spider silk	Bolt Threads (US)
		Secondary metabolites: Antibiotics	Pfizer (US) Merck (US)
		Flavourings and fragrances	Gingko Bioworks (US) Amyris (US)
		Bioplastics (polyhydroxyalkonoate)	Metabolix (Yield10 Biosciences) (US)
Yeasts: *Saccharomyces cerevisiae*, *Pichia pastoris, Yarrowia lipolytica*	Carbon: starch and cellulosic biomass Nitrogen: ammonium/nitrate salts, soy protein, amino acids, urea, yeast extract Mineral and trace elements	Therapeutic proteins: insulin	Genentech (US) Eli Lilly (US) Novo Nordisk (Denmark)
		Industrial and molecular enzymes	Novozyme (Denmark) DuPont (US) DSM (Netherlands) Codexis (US) AB Enzymes (Germany)
		Secondary metabolites: Vanillin (flavour and fragrance compound) Stevia (non-calorific sweetener) Resveratrol (health product) Artemisinin (anti-malarial drug precursor)	Evolva (Switzerland) Conagen (US) Gingko Bioworks (US) Amyris (US)
		Bioethanol	POET (US) Valero (US) Abengoa (Spain)
		Omega-3 fatty acids	DuPont (US)
Mammalian cell lines: Chinese Hampster Ovary (CHO), Hybridoma and other cell lines	Glucose, L-glutamine, sodium pyruvate, mineral salts. Foetal bovine serum/recombinant growth factors are also commonly added.	Erythropoietin Monoclonal antibodies Antibody-drug conjugates Vaccines Industrial enzymes Biomolecules for molecular research	Amgen (US) Genentech (US) New England Biolabs (US)
Insect cell lines (Sf9, Sf21, High Five, S2, etc.)	Tryptose phosphate broth, yeast extract, L-Glutamine, mineral salts. Optionally: insect haemolymph, foetal bovine serum/recombinant growth factors	Vaccines, therapeutics, diagnostic reagents, growth factors, gene therapy adenovirus production, industrial enzymes, biomolecules for molecular research	Novavax (US) Oxford Expression Technologies (UK) Protein Sciences Corporation (US) GSK (UK) Dendreon (US) UniQure (Netherlands) Novimmune (Switzerland)

**Table 2:** Performance characteristics of example whole insects commonly used for commercial purposes

Organism	Substrate	Typical Feed conversion ratio (FCR)	Common Products	Commercial examples
Cabbage looper (*Trichoplusia ni)*	Agar, wheat germ, cornmeal, soybean meal, casein, soybean oil, vitamins and salts	Data not available	Vaccines, therapeutics, diagnostic reagents, growth factors, enzymes for molecular biology	Algenex & Cocoon Biosciences (Spain) Chesapeake PERL Inc. (US), Entopath (US)
Honey Bee (*Apis mellifera*)	Flower nectar and pollen	Data not available	Honey, beeswax	Various: Comvita (NZ), Dutch Gold Honey (US), Rowse Honey (UK) Capilano Honey (Australia), etc.
Silkworm (*Bombyx mori*)	Mulberry leaves	7.3–7.967^167^	Silk production, pet food, livestock feed, aquaculture feed Protein therapeutics Spider silk	Wujiang First Textile Company (China), Sericulture Industries (India). Katakura (Japan) Kraig Biocraft Laboratories (USA)
Black soldier flies (*Hermetia illucens*)	Organic waste from fruits, vegetables, grains, meat and manure	~1.4–2.6^168^ Also see ref. ^169^ for FCRs across broader organic waste streams.	Pet food, Livestock feed, Aquaculture feed	AgriProtein (South Africa), Innovafeed (France), Protix (Netherlands), Enterra (Canada), Protenga (Malaysia), Nutrinsecta (Brazil), Goterra (Australia).
Crickets and grasshoppers (*order Orthoptera)*	Organic waste from fruits, vegetables and grains	~2.3–10^168^	Human and pet food, livestock feed, aquaculture feed	Aspire Food Group (US), Entomo Farms (Canada), Bugsolutely (Thailand), Micronutris (France)
Meal worms (*Tenebrio molitor*)	Organic waste from fruits, vegetables, grains and manure	~3.8–6.1^168^	Pet food, livestock feed, aquaculture feed, fish bait	Ÿnsect (France), Rainbow mealworms (US), Grubco (US), Enterra (Canada), ProBugs (Netherlands)
House fly maggots (*Musca domestica*)	Organic waste from fruits, vegetables, grains, meat and manure	Data not available	Pet food, Livestock feed, Aquaculture feed	AgriProtein (South Africa), Bioflytech (Spain), Entomics Biosystems (UK)
Superworm beetle larvae (*Zophobas morio)*	Organic waste from fruits, vegetables and grains	Data not available	Pet food, Livestock feed, Aquaculture feed	Rainbow mealworms (US)
Wax worms (*Galleria mellonella*)	Organic waste from beeswax, fruits, vegetables and grains	Data not available	Pet food, livestock feed, aquaculture feed, fish bait	Waxworm Farm (US), Grubco (US), Timberline Fisheries (US)

## BSF biomanufacturing for the valorisation of organic waste

Insect pupae biomanufacturing platforms, including the cabbage looper (*T. ni*), are already used to commercially biomanufacture biomolecules^51,59^. However, these insects cannot be reared on organic waste and their relatively narrow dietary requirements^60^ can hinder scalability. Adoption of insect species with an appetite for a variety of organic wastes have the greatest potential for contributing to sustainable biomanufacturing on a global scale.

Many insect species are adept at decomposing organic waste and perform an important ecosystem service to recycle nutrients. Black soldier flies, (BSF; *Hermetia illucens*), house fly maggots (*Musca domestica*), crickets and grasshoppers (*order Orthoptera)*, superworm beetle larvae (*Zophobas morio)*, meal worms (*Tenebrio molitor*) and wax worms (*Galleria mellonella*) have all been adopted commercially for their strengths in processing organic waste into insect protein (Table 2).

BSF especially have gained interest as a rapid means to convert large quantities of organic wastes into a variety of products (Table 2), as many aspects of their biology and lifecycle readily support large-scale rearing practices in commercial settings. They are not considered a pest species and they are globally distributed, except for Antarctica^61^. Furthermore, they are not a carrier for human pathogens as they do not feed on waste as mobile adults and do not bite^62^. BSF larvae (BSFL) can consume up to 500 mg of organic matter per day per larvae^55^. Optimal rearing conditions for BSF can be applied within rearing facilities and is largely influenced by temperature, humidity, substrate composition, among other conditions^63^; it is an active area of BSF research^64^. BSFL thrive on a wide variety of organic wastes including kitchen waste, fruits and vegetables, meat and fish, livestock manure and even human excrement^64^. The insects are easily harvested from the processed organic waste with a sieve or when the larvae migrate out of the organic waste to pupate^65^. Typically, on a dry weight basis, the pupae contain 40–44% protein and 15–50% lipid^55^. The composition of BSFL, particularly the lipid fraction, can vary considerably depending on the lipid composition of the substrate mixtures they consume as larvae^66^. The main products made from BSFL/pupae are pet food and livestock feed^54,67^. Though BSFL/pupae have also been used to make other value-added products including biofuels^68^, bioplastics^69^, lubricant additives^70^, antimicrobial compounds (AMPs)^71^, chitin and chitosan^72^, sustainable protein for human consumption^73^, among other applications^74^.

BSF is used in waste management to reduce the quantity of organic waste, with waste reduction efficiencies between 65.5% and 78.9% under conditions that rival standard composting methods and on a timescale of weeks compared to months^67^. The processed organic waste, insect exuviae and associated microbes, are referred to as frass which is used as an effective soil amendment to recycle nutrients, such as nitrogen and phosphorous, thereby promoting plant growth^75^.

## Engineering BSF for biomanufacturing

Utilising transgenic BSF to support a sustainable circular economy involves considering both waste streams and products. Waste-streams may be broadly divided into two categories: those that are suitable to produce food/feed and those that are not. The former high-grade wastes includes horticultural, food manufacturing, fisheries and pre-consumer food waste^76^. The latter includes low-grade wastes such as restaurant and post-consumer household organic waste, slaughterhouse waste, livestock manure, municipal biosolids and organic waste contaminated with hazardous compounds. The suitability of a particular waste stream to be used as feed will depend on safety, public perception and regulatory factors^76^.

**Fig. 1:**
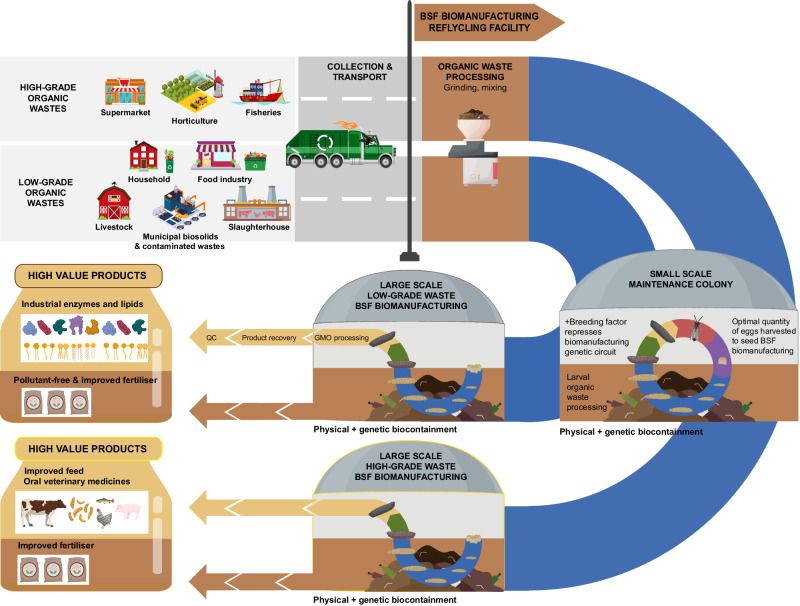
Engineering BSF for industrial biomanufacturing and bioremediation. BSF biomanufacturing facilities could valorise a broader variety of organic wastes into high-value products such as improved feed, oral therapeutics, industrial biomolecules such as enzymes and lipids and clean high-quality frass. Figure created using Adobe Illustrator, Adobe Stock Images under an education license and Biorender.com.

A major limitation of using wild-type BSF to generate predominantly animal feeds from waste are regulations which preclude using low-grade organic wastes to rear BSF for feed^76^. BSF could, however, valorise both high- and low-grade wastes by engineering BSF as a platform for biomanufacturing and bioremediation (Fig. 1). BSF could convert greater varieties of low-grade organic wastes into insect biomass engineered to biomanufacture a large variety of high-value biomolecules such as industrial enzymes and lipids, that are not subject to food/feed regulations. Engineered BSF could also convert high-grade organic wastes into enhanced animal feeds. Furthermore, there is opportunity to generate enhanced fertiliser from both high- and low-grade organic wastes.

## Valorising high-grade organic wastes

As previously discussed, BSF are becoming widely used to produce animal feed and animal feed ingredients^54,55,67^. The value of these ingredients could be increased by engineering BSF to express enzymes that are commonly added into feed ingredients. Amylases, pectinases, lipases, proteases and phytases are currently made by microbial fermentation and incorporated into animal feeds to boost digestion of other ingredients^77^. These enzymes enable the use of less expensive feed ingredients, increase growth rates and in the case of phytases—also reduce phosphorous waste in manure. BSF engineered to express these enzymes may additionally improve their own FCR on otherwise difficult to digest organic waste feedstocks. Altering the lipid profile may be desirable by, for example, engineering BSF to produce marine-derived omega-3 fatty acids^78^ which would improve the fatty acid profile of aquaculture animals reared using BSF. BSF engineered to improve feed for these purposes is not likely to encounter significant regulatory hurdles as many genetically engineered products are already approved for use as feed^77^. For example, food crops genetically engineered to improve productivity in the field are inexpensive sources of animal feed^79^ and enzyme supplements used to improve digestibility are sourced from genetically engineered microbes^77^.

It may also be possible to engineer BSF to produce oral veterinary medicines and vaccines for livestock^80^. BSF is already palatable for livestock rearing which may enable these veterinary medicines to be administered easily at scale^73^. Moreover, they act as a pre-biotic to promote a healthy and diverse microbiome^81,82^. BSF could improve feed and food safety further by engineering BSF to prevent the growth of harmful microbes^83^ and to remediate chemical pollutants found in pre-consumer waste such as heavy metals^84^, biocides^85^ and mycotoxins^86^. Currently, therapeutic vaccines for rabbit haemorrhagic disease are already produced by genetically engineered cabbage looper pupae^87^, which have marketing authorisation in the European Union and United Kingdom^88^. Expanding to BSF is therefore likely to be possible from both technical and regulatory perspectives.

## Valorising low-grade organic wastes

Transgenic BSF should also be capable of valorising waste streams not suitable for feed due to hygiene or chemical contamination regulations. These could be used as feedstocks to produce high-value industrial biomolecules, which are not subject to hygiene considerations required for feed or food consumption^76^. This includes enzymes such as laccases, amylases, hemicellulases, cellulases, proteases, peroxidases and lipases which have applications in textile processing, feedstock pre-treatment for biofuels, paper manufacturing and as ingredients in detergents^89^. Enzymes could also be biomanufactured by BSF to clean up industrial wastewater. These enzymes may be produced from BSF in crude or purified form, lyophilised for ease of storage and transport and sold to industry.

For biofuels, extracellular carbohydrase and ligninolytic enzymes can be initially extracted in aqueous buffers to provide an economical enzymatic pre-treatment method for processing complex lignocellulose feedstocks into fermentable sugars for industrial microbes. The BSF lipid fraction could then be extracted from the insoluble fraction and processed into biodiesel^68^. The protein in the remaining solid phase fraction could be fed to industrial microbes as their nitrogen source for biofuel production.

Beyond valorising wastes that BSF can currently consume, BSF could be engineered to express enzymes that can increase the scope of wastes they are able to process. This could include agricultural lignocellulosic wastes^90^, fats/grease^88^, untreated sewage^91^ and chemically contaminated wastes^8–10,92^. Soiled mixed plastic waste could also be used as a feedstock, though its mineralisation will generate net-positive CO_2_ emissions. This could potentially be offset if the BSF generate other biomolecules with high carbon content or traditionally produced via fossil fuels e.g. for generating bioplastics^69^, industrial lipids^93^, or carbon polymers for carbon credits. Alternatively, BSF could generate lyophilised enzymes for the plastic recycling industry, to regenerate the starting monomers from plastic waste^94,95^.

BSF could be engineered for organic waste bioremediation to clean up and redeem value to organic wastes that are chemically contaminated. This could include 25% of all food wastes contaminated by mycotoxins^86^ and chemical pollutants typically found in biosolids such as heavy metals and PFAS^8,10,96^. Contaminated soil could additionally be mixed into organic waste in optimised ratios as a potential ex vivo bioremediation service^97^. BSF may be engineered to achieve bioremediation via enzymatic catabolism or potentially via engineering the larvae to hyperaccumulate contaminants and leave behind clean frass for fertiliser.

We demonstrated a proof of concept for engineering insects for biomanufacturing and bioremediation by engineering the model insect *Drosophila melanogaster* to produce and secrete a functional fungal laccase from *Trametes trogii*^98^. Fungal laccases are powerful enzymes with a broad substrate range and they have demonstrated utility for a variety of applications. For example, lyophilised laccase could be made from BSF pupae for their use in the textile^99^, paper and pulp^100^, food^101^, pharmaceutical^102^, chemical synthesis^103^ and forestry^104^ industries. Furthermore, they can biodegrade lignin in lignocellulose and certain plastics^105,106^ and may allow these organic wastes to now be processed by the BSFL. They can also be used for bioremediation to clean up contaminants in organic waste and make clean frass; for example, pollutants such as bisphenols^107^, PFAS^108,109^, mycotoxins^110^, microplastics^105^, biocides^111^, pharmaceuticals^112^, among others. We demonstrated that engineered *D. melanogaster* was capable of degrading laccase substrates within their diet, including bisphenol A (BPA), while a lyophilised powder made from adult flies could decolourise the dye indigo carmine for industrial wastewater treatment^98^. Approximately 200,000 tons of dye is lost to textile industry effluents each year^113^. In waterways, dyes hinder light penetration, which affects photosynthesis and oxygen availability^113^. These effluents could potentially be economically treated by lyophilised laccase generated by BSF.

We additionally demonstrated that *D. melanogaster* could be engineered to express the microbial enzymes, organomercurial lyase (MerB) and mercuric reductase (MerA) from *E. coli*, to bioremediate methylmercury^114^. The engineered insects exposed to methylmercury in their diet were able to demethylate methylmercury and remove it from their biomass as atmospheric elemental mercury. Currently, a quarter of the mercury inventory that is released into the environment is from municipal sewage^115^. BSFL could be engineered to express MerA and MerB to clean up and restore value to organic wastes contaminated with mercury, such as municipal^115^ or fisheries^92^ organic wastes. The atmospheric elemental mercury can be trapped^116^ in physically contained facilities and safely stored away from the biosphere. Meanwhile, the clean insect biomass could be used to biomanufacture high-value biomolecules or animal feed and the clean frass can be maintained as fertiliser for crop growth.

## Improved fertiliser

Increased BSF production and generation of frass could reduce our reliance on synthetic fertilisers. Synthetic fertiliser manufacturing accounts for 2.5% of global CO_2_-equivalent of annual GHG emissions^117^. This statistic does not account for the quantity of N_2_O volatilisation once it is applied to the soil, which is a GHG that is 300-times more potent than CO_2_^117^.

From a soil science perspective, frass provides several additional advantages over synthetic fertilisers. The insect exuviae found in frass stimulate plant defences and may attract beneficials to promote plant health^75,118^. Synthetic fertilisers notoriously cause nutrient runoff and waterway eutrophication. Frass can retain phosphorous and potassium while maintaining absorption by plants and may reduce eutrophication from runoff^75,119,120^. Meanwhile, frass can improve healthy microbial diversity in the soil^121^. Microbes present in organic compost temporarily incorporate nitrogen from the soil into the amino acids of their proteins, preventing runoff of soluble nitrogen species^122^. As the microbes exhaust the nutrients in the fertiliser and microbial populations decline, this provides plants with slow-release nitrogen that promotes root growth.

BSF could be engineered to improve the quality of frass for crop outcomes. BSF could be engineered to improve arable soil; for example, through secreting enzymes or binding molecules into the frass that can remediate a contaminant or improve other biotic or abiotic conditions in the target site^123^. Soil is also increasingly recognised as a powerful sink for carbon capture^124^ and with economic policies that reward carbon capture, BSF could be engineered as a commercial carbon capture option.

## Advantages and disadvantages of BSF biomanufacturing

BSF have several advantages over industrial bioreactor-based biomanufacturing platforms traditionally used to produce high-value biomolecules. Industrial bioreactor-based biomanufacturing requires feedstocks that provide carbon and nitrogen (e.g. sugar and amino acids), enzyme co-factors, growth factors (e.g. foetal bovine serum for mammalian/insect cell culture) and other mineral and trace nutrients^31^. These can be sourced from a variety of resources ranging from petrochemicals and food crops to waste by-products^31^ and a full life-cycle assessment is necessary to evaluate the relative sustainability of each. Regardless of the source, these ingredients must be processed via resource and energy intensive mechanisms. Currently, carbon in large scale biomanufacturing is largely derived from food crops, which is typically processed by energy intensive milling and grinding and juice extraction and heating during clarification. Additional chemical, thermal and enzymatic pre-treatments may be required for waste feedstocks such as lignocellulose^125^ and certain microbial strains are only able to metabolise hexose or pentose sugars for fermentation^126^. Nitrogen is typically sourced from inorganic ammonia made in the fossil fuel intensive Haber-Bosch process^31^. Notable trace elements like phosphorous is unsustainably mined using energy-intensive practices^31^. Moreover, sterilisation in some applications may be necessary to remove competing microorganisms that may reduce the product yield.

BSF are able to derive carbon, nitrogen and trace elements like phosphorous from the wide variety of organic waste they are able to consume. Minimal organic waste feedstock pre-processing is required as animal digestion is highly evolved to extract and absorb nutrients: food is physically broken into smaller pieces by mastication, the digestive tract consists of several compartments with acidic and alkaline regions and various digestive enzymes process biopolymers into components that can be absorbed along with other essential nutrients^127^. Furthermore, additional chemical energy from complex variable dietary fibre from a diversity of plants can be broken down by the diverse microbiome in the insect hind-gut to generate short chain fatty acids that can be absorbed and utilised by the insect host^128^.

BSF do not require thermally treated or sterilised inputs as they have evolved excellent tolerance to their fellow organic composters such as bacteria and yeast^128^. These organisms may digest feedstocks that are difficult to process, which can in turn be consumed by BSFL^128^. However, BSF are susceptible to entomopathogenic bacteria, viruses, fungi, nematodes, protozoa, parasitoids, or mites^129–131^. Unexplained colony collapse has been reported for BSF colonies (unpublished) and further research is required to determine whether this is due to abiotic and/or biotic causes.

Microbial biomanufacturing requires sophisticated infrastructure and highly skilled personnel which are significant barriers to adoption by low-income countries. This includes oversight by scientists and engineers and infrastructure for feedstock extraction and quality assurance; equipment sterilisation; fermentation; significant water purification facilities; waste management; heating and cooling; control and monitoring; and downstream genetically modified organism (GMO) processing, product extraction, purification and quality assurance.

Though industrial BSF farming can be automated with sophisticated infrastructure^132^, it is not required and can be adopted and maintained readily worldwide^133^. BSF facilities that house genetically engineered strains however, will need to design/upgrade their facilities and train personnel to comply with biocontainment standards relevant to the country. An overview of a potential BSF biomanufacturing facility is shown in Fig. 1. The benefit of housing engineered strains in physically contained facilities is that it will avoid onerous regulatory hurdles required for intentional release of transgenic organisms into the environment, such as what is required for transgenic plants^34,35,134^. Similar to microbial biomanufacturing, the products made by transgenic BSF can be removed from the contained facility after the insects are treated with a ‘physical or chemical process which removes, kills or renders non-viable the GMOs used’^134^. For example, freezing, microwaving, baking, boiling, blanching, desiccation, grinding, asphyxiation, or high hydrostatic pressure can be used to kill black soldier flies^135^. Genetic biocontainment strategies could be stacked for additional security. Genetic biocontainment has been demonstrated in insects to prevent interbreeding with their wild counterparts^136^, as well as strains that cannot fly^137^ and would incur a significant fitness cost in the wild but not within the facility. Genetic biocontainment strategies will be discussed further in the ‘Biocontainment’ section below.

BSF is likely to be regulated similarly to other GMO biomanufacturing platforms, with the exception that products intended to make feed or food products would need to be regulated for feed or food production. Numerous modern products are available on the market in Europe, Asia, Australia, Africa and the Americas that are derived from GMOs. These range from food and feed derived from engineered crops^79^ to vitamins^138–140^, enzymes^77^, polymers^141^ and pharmaceuticals^24,26,142^ produced by microbial fermentation. Moreover, BSF is approved for feed or food production using high-grade organic wastes in the European Union, Asia, Africa, Australia and the Americas^76^.

Though single cell organisms have a fast turnaround for generating results rapidly for research and development, scaling microbial industrial biomanufacturing continues to be challenging, particularly at the fermentation stage^143^. BSF farming is already scaling considerably, with BSF facilities growing in number across the globe. One of the largest industrial BSF facilities, AgriProtein in South Africa, processed 250 tonnes of organic wastes per day and generates 7 tonnes of larvae meal, 3 tonnes of oil and 20 tonnes of fertiliser daily^144^. Enterra in Canada processes 100 tonnes of pre-consumer organic waste per day and produces 7 tonnes of protein and oil feed ingredients and 8 tonnes of fertiliser per day^144^. Engineering BSF as a biomanufacturing platform for high-value biomolecules could therefore be readily applied at scale, building on this existing infrastructure. Though not directly comparable, according to Capacitor data, globally there are 20 microbial bioreactor facilities in the range of 20,000–99,999 L capacity and 9 bioreactor facilities with greater than 100,000 L capacity^145^. Large companies such as Genentech use bioreactor-based fermentation to make 10 metric tonnes of biologics annually^146^.

A major bottleneck for BSF biomanufacturing is that the genetic toolkit for BSF is yet to be as developed as other industrial biomanufacturing platforms. The first BSF genome was published in 2015^147^ and has since been sequenced by other groups^137,148^. There are currently four reports in the literature describing heritable BSF genetic manipulation: one involving Cas9 gene knock-outs^137^ and our research group and two other research groups have demonstrated piggyBac transposase mediated random chromosomal integration^149–151^. Though commercially valuable products could currently be generated using piggyBac transposase-mediated integration for one or two transgenes, random chromosomal integration complicates strain development and strain performance. It results in variable transgene expression levels due to positional effects and frequently results in the integration of multiple transgene copies. Uncertainty over the location and copy number of a transgene creates challenges for establishing homozygous strains and some genetic manipulations benefit from integration in sex-chromosomes. Further research will be required to demonstrate heritable locus-specific genome integration methods to address these challenges, which will be discussed in more detail in the ‘Research and Genetic Tools’ section. Further, into the future, more complex metabolic engineering will be required. Design considerations for genetic and metabolic engineering from other organisms will speed up its implementation in BSF.

## Addressing the sustainable development goals (SDGs)

The SDGs are a set of 17 objectives established by the United Nations (UN) to address the world’s most pressing challenges before 2030^30^. Broadly, these goals provide a framework to address issues from poverty and inequality, to environmental degradation and peace. BSF biomanufacturing as showcased here could address multiple SDGs (Table 3) for a more sustainable and equitable future.

**Table 3:** BSF Biomanufacturing and the SDGs

SDG(s)	Addressing the SDGs with BSF Biomanufacturing
SDG #1: End poverty in all its forms everywhere	• BSF biomanufacturing will offer economic opportunities and could be adopted worldwide to valorise widely available and underutilised organic wastes.
SDG #2: End hunger, achieve food security and improved nutrition and promote sustainable agriculture	• Compared to other biomanufacturing methods, BSF biomanufacturing does not require feedstocks derived from food crops and will improve food security. • BSF biomanufacturing for generating value-added animal feeds will improve nutrition and livestock productivity. • Greater adoption of BSF biomanufacturing will increase frass production for improved crop growth.
SDG #3: Ensure healthy lives and promote well-being for all at all ages	• In 2015, pollution was the leading environmental cause of premature death and disease globally^170^. BSF biomanufacturing for the generation of high-value products could clean up pollutants in organic wastes and reduce open dumping and burning of organic wastes.
SDG #6: Ensure availability and sustainable management of water and sanitation for all	• BSF biomanufacturing for the generation of high-value products would incentivise the development of infrastructure to collect organic waste, reducing open dumping and the consequent proliferation of pests and contamination of waterways. • BSF biomanufacturing could generate products that can inexpensively clean up industrial wastewater. • BSF farming requires significantly less water than livestock and horticulture^73^.
SDG #8: Promote sustained, inclusive and sustainable economic growth, full and productive employment and decent work for all	• BSF biomanufacturing provides exciting job opportunities for researchers and entrepreneurs that will help to solve some of the world’s most pressing problems.
SDG #9: Build resilient infrastructure, promote inclusive and sustainable industrialisation and foster innovation	• BSF biomanufacturing would provide an opportunity for the private sector to valorise organic waste and develop the infrastructure required to collect and utilise organic waste. • BSF biomanufacturing is not yet established and provides many opportunities for research and innovation.
SDG #11: Make cities and human settlements inclusive, safe, resilient and sustainable SDG #12: Ensure sustainable consumption and production patterns	• BSF biomanufacturing could improve biomanufacturing sustainability and slow climate change, reduce pollution and eutrophication and reduce organic waste burning and improve air quality.
SDG #13: Take urgent action to combat climate change and its impacts	• The waste sector is estimated to be responsible for 5% of global annual greenhouse gases^6^, mainly due to landfilling organic wastes. BSF biomanufacturing will reduce organic waste landfilling. • Synthetic fertilisers are estimated to be responsible for 2.5% of annual greenhouse gas emissions^117^ and this does not include the volatilisation of the potent greenhouse gas, N_2_O, from synthetic fertiliser applied to soils. Greater adoption of BSF biomanufacturing and production of frass could reduce our reliance on synthetic fertilisers. • Agriculture and its associated land use are estimated to be responsible for 17% of total annual greenhouse gas emissions^117^. Improved animal feeds will improve livestock productivity. BSF engineered to improve frass could improve horticulture productivity. • BSF biomanufacturing does not require feedstocks from food crops and will reduce land clearing and preserve forests as a large carbon sink. • BSF could be engineered as a platform for soil carbon capture for carbon credits.
SDG #14: Conserve and sustainably use the oceans, seas and marine resources for sustainable development	• BSF engineered to synthesise marine omega-3 fatty acids could reduce the requirement for wild-caught fish feed to support adequate aquaculture nutrition. • BSF engineered as a platform for biomanufacturing and bioremediation will reduce pollutants and organic waste eutrophication in the environment and improve species conservation. • BSF engineered to process soiled mixed plastic wastes will allow plastics to be processed locally and reduce plastic pollution in the ocean.
SDG #15: Protect, restore and promote sustainable use of terrestrial ecosystems, sustainably manage forests, combat desertification and halt and reverse land degradation and halt biodiversity loss	• BSF biomanufacturing could reduce land clearing for crops and thereby preserve forests, habitats and biodiversity: 1) Compared to other biomanufacturing methods, BSF biomanufacturing does not require feedstocks derived from food crops. 2) BSF frass could improve soil quality and improve crop productivity per unit of land. • BSF biomanufacturing could halt biodiversity loss: 1) Climate change and pollution are major threats to species conservation^171^ and BSF engineered as a platform for biomanufacturing and bioremediation could address significant sources of emissions, as well as reduce pollution in the environment.

## Establishing BSF biomanufacturing

**Fig. 2:**
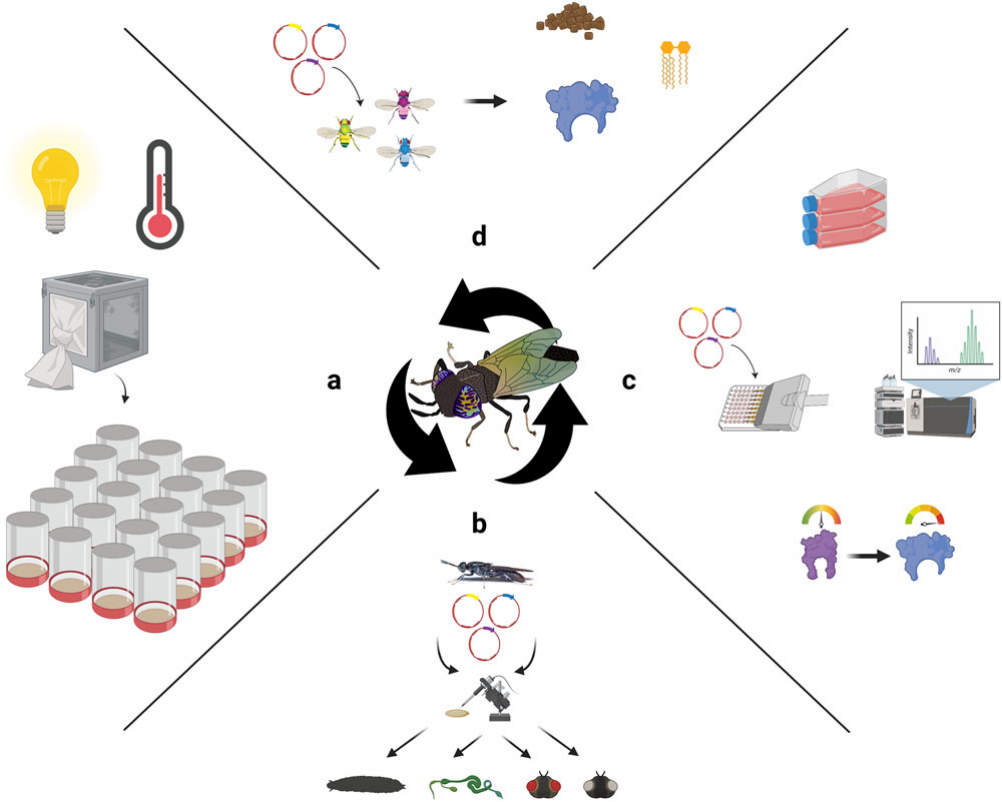
Developing the BSF synthetic biology platform. **a** Optimising lab-scale rearing protocols to minimise space requirements, simplified media formulations and tools for long-term storage. **b** Streamlining transgenesis protocols and characterising genetic parts such as tissue specific and small molecule regulated promoters. **c** Establishment of high-throughput enzyme testing and directed evolution capabilities in BSF cell culture. **d** Establishing the optimal expression patterning in the insect genetic model, *D. melanogaster*. Figure created using Adobe Illustrator, Adobe Stock Images under an education license and Biorender.com.

The critical next steps for realising BSF manufacturing will involve first developing the research and genetic tools for BSF as a synthetic biology and biomanufacturing platform (Fig. 2). It will also involve identifying functional candidates for high-value biomolecules that are appropriate to the strengths of insect biomanufacturing and developing BSF strains that can process a greater scope of organic wastes. These could be licensed to BSF facilities as stable germline (heritable) transgenic strains. BSF facilities will need to ensure their facilities and staff comply with physical containment standards for housing the genetically engineered insects. BSF facilities will also be required to obtain regulatory approvals. BSF biomanufacturers can then begin to scale up organic waste collection appropriate to the type of biomanufacturing. It will be critical for both BSF strain developers and BSF facilities to establish relationships with downstream industries to ensure BSF products will ultimately be used.

## Research and genetic tools

Substantial effort is required for BSF to catch-up to more established research strains and biomanufacturing platforms. Scientific research into BSF synthetic biology as well as basic research into their biology (for example investigating colony collapse, improving fertility, optimising the proportion of protein or lipids, investigating overwintering mechanisms, studying microbiome interactions, etc.) is required to maximise their utility as a biomanufacturing platform. This will require establishing lab-scale research tools for BSF husbandry to enable high-throughput scientific testing for multiple test conditions and simplifying maintenance for multiple strains (Fig. 2a). This will include scaling down rearing containers, establishing simplified low-odour media formulations and developing tools for long-term storage.

It will be critical to establish robust and efficient genetic engineering tools in BSF to establish heritable transgenic lines (Fig. 2b). Genetic engineering, particularly for multicellular eukaryotic organisms, is complex and requires a highly specialised skillset and equipment^152^. While genetic engineering has been described in BSF using random chromosomal integration methods^149^, further research is required to develop efficient locus specific chromosomal integration methods. This will facilitate the characterisation of copy number and positional effects and allow for the easier generation of homozygous lines to maintain stable transgenic BSF strains. Such advancements can be achieved with technologies such as CRISPR-Cas9 or PhiC31 integrase-mediated transgene integration^152^. A PASTE system was also described recently that uses prime-editing to add a bacteriophage AttP landing site into a target locus and simultaneously integrate a target genetic construct^153^. These tools could be used in BSF to insert constructs into chromosomal regions known to be in euchromatin for the desired expression patterning, or to disrupt genes that are required for fitness in the wild, but not within a biomanufacturing facility^137^.

Generating strains with well-characterised PhiC31 AttP landing sites that are broadly available proved very useful for *D. melanogaster* research, where they are frequently used by commercial transgenesis services^154^. These services have allowed new frontiers in *D. melanogaster* research to rapidly progress as the complex transgenesis procedures can be outsourced by well-equipped professionals^154^. Similar commercial providers for BSF will allow widespread adoption of research into BSF synthetic biology.

Further research is required to characterise regulatory elements (promoters, untranslated regions (UTRs), and terminators) that can be used to express transgenes at different strengths ubiquitously; inducibly; in specific tissues such as the salivary gland, midgut, hindgut, haemolymph, fat body, or casings; or at specific developmental stages such as during larvae development, pupation, or adulthood (Fig. 2b). Prior research from other insects/organisms or investigating BSF entomopathogenic viruses may reveal constitutive (e.g. from endogenous housekeeping genes, or strong viral promoters) and inducible promoters (e.g. nutrient, chemical, thermal, or light sensing promoters). Inducible promoters have been established in insects for utilising antibiotic responsive promoters^155^ or plant hormones, such as auxin^156^, which are inexpensive to make at industrial scales. Prior transcriptomics studies in BSF may guide tissue- and developmental-stage^157^ specific promoter discovery before validating these promoters by using RT-qPCR and/or colorimetric/fluorometric reporter fusion constructs.

Biodegradative enzymes that will be required for enhancing waste processing could be expressed specifically during the larval life-stage from a larval promoter. Meanwhile, enzymes required for bioremediation could be expressed from pollutant sensing inducible promoters or at all life-stages. Target high-value biomolecules could begin to be overexpressed in all cells specifically during pupation, when the insects are separated and collected, to maximise target biomolecule yield while minimising a fitness cost to the larvae during waste processing (Fig. 1).

## Prototyping insect biomanufacturing

Heterologous expression of microbial, algal, fungal, and plant enzymes in a distantly related insect host can be difficult to achieve as enzymes adapt to function in the conditions of their native host. An enzyme may not be functional in a new host due to differences in cellular chemistry or cellular machinery. This could include issues with cellular redox potential, pH, oxygen sensitivity, as well as problems with protein solubility, misfolding, post-translational modifications, localisation, pre-pro-peptide processing and the requirement for chaperones. These issues can be difficult and time consuming to troubleshoot. Moreover, an enzyme may be toxic to the animal host, which would also preclude their suitability. As there is a vast library of useful enzymes and proteins characterised to date, libraries of enzymes and proteins can be screened and it is likely at least some will be active and will not cause harm.

BSF cell lines (Fig. 2c) or the insect genetic model, *D. melanogaster* (Fig. 2d), could serve as initial test platforms to rapidly screen libraries of enzymes and proteins. BSF cell lines could be developed to rapidly screen for enzyme function and then many hundreds of variants can be generated for directed evolution to optimise enzyme activity in the conditions of the intended downstream application (for example, in the BSF digestive tract, in the digestive tract of livestock/aquaculture, in the downstream industrial setting, etc.)

*D. melanogaster* could be used to optimise an enzyme or protein’s spatial and temporal expression patterning. *D. melanogaster* is closely related to its dipteran cousin, BSF and enzymes and proteins that are active in *D. melanogaster* are likely to also be active in BSF. *D. melanogaster* will provide a rapid turnaround of results as it has a short generation time of 10 days at 25 °C, high fecundity where one female can produce >75 eggs per day^158^ and it is simple and inexpensive to maintain. Its genome is thoroughly characterised and its genetic engineering can be reliably outsourced commercially for multiple genomic loci with known positional effects^154^. Well characterised balancer chromosomes also enable the rapid generation of stable homozygous transgenic stocks^159^. Experimental designs can be rapidly adjusted in BSF cell lines and *D. melanogaster*, and these validated experimental designs can be readily applied to functional assays in BSF.

Optimised enzyme variants with optimal expression patterning could then be engineered into insects, such as BSF, that are well suited for biomanufacturing, but might not be optimal as a research tool.

Recently, ref. ^160^ reviewed how *D. melanogaster* could be an excellent bioreactor for plastic degradation^160^. They list microbial, fungal, insect and algal enzymes that are able to degrade different types of plastic and describe what spatial and temporal expression patterning could be used to express these enzymes in *D. melanogaster* for plastic degradation. They also describe how the microbiome in the insect hind-gut could be altered with plastic degrading microbes or microbial communities to synergistically break down plastic.

For the purpose of BSF biomanufacturing, the technology readiness to alter an animal’s microbiome is not as established as genetically engineering an animal. To date, it remains very difficult to stably alter an animal’s microbiome, as these microbes face fierce competition from the native microbiome^161^, which are well adapted to survive in the intestinal mucosa and derive energy from indigestible nutrients commonly available in the lower gastrointestinal tract^128,162^. It would therefore require constant selection, and effective transgene biocontainment strategies for the microbiome are not yet established^163,164^. It would potentially also require expensive sterilisation of the organic waste feedstocks. A diverse microbiome performs a service to the host to degrade indigestible fibre from a diversity of plants, as well as prevent colonisation of harmful bacteria^162^. Significantly altering BSF’s native microbiome may impact BSF’s ability to process a wide variety of organic waste feedstocks^128^. Though it is challenging to alter an animal’s microbiome, it may be possible to develop microbial pre-treatments to improve BSF digestibility of challenging substrates^90^. Research has shown that pre-treatment of lignocellulose feedstocks with microbes and fungi can improve the bioconversion of lignocellulose by BSF^90^. Nevertheless, implementing pre-treatments is challenging at industrial scales^90^ and directly engineering BSF to express the required degradative enzymes would mitigate the necessity for pre-treatments.

## Biocontainment

The risk landscape for this technology is not yet fully understood and biocontainment strategies and transparent risk assessments are required to reveal how this technology can be used to maximise benefit and minimise harm. Though unanticipated risks are by definition not predictable, unintended risks could include escape of transgenic BSF, that are now able to exploit a broader scope of food sources, disrupting ecosystems or leading to the evolution of new pest species. Fungal laccases are able to degrade pesticides^111^ and escapees engineered to express these enzymes may be difficult to eradicate. It is notable however that no genetically engineered microbial strain from a physically contained facility has escaped or caused known harm^165^.

The main biocontainment strategy to prevent transgenic BSF escape will be to rear BSF in physically contained facilities^134^. This strategy is widely used to date without issue for a variety of transgenic organisms from microbes to animals in both research and industrial settings^165,166^. Additional genetic biocontainment strategies could also be stacked for greater security. For example, by developing strains that cannot reproduce with their wild counterparts^136^ and disrupting genes essential for survival in the wild but not in the contained composting facilities (e.g. flight, sight, eclosion etc.). Silkworms are an example of domesticated insects that cannot survive in the wild as they can only subsist on mulberry leaves and have now lost the ability to fly. Flightless BSF has been demonstrated by using CRISPR-Cas9 knock outs^137^. Moreover, survival could be regulated by synthetic auxotrophies to make strains dependent on compounds not available out of the contained facilities^165^. These tools have been demonstrated in various single cell organisms^165^. This could be achieved by engineering BSF with a repressible biomanufacturing genetic circuit. In the absence of the repressor, the biomanufacturing genetic circuit switches global gene expression from the pre-pupae life-stage onwards to favour only genes required for high-yield target biomolecule overexpression. All other genes are not expressed and the host is thereby rendered non-viable (Fig. 1). The repressor for the biomanufacturing genetic circuit could be supplied in the contained facilities for the maintenance of breeding colonies.

## Conclusion

BSF is a promising synthetic biology platform to improve sustainability in biomanufacturing and allow greater valorisation of organic wastes. To turn this idea into a reality, further research is required to genetically characterise BSF as a synthetic biology platform and to develop BSF biomanufacturing strains that can produce high-value biomolecules on a broader scope of organic wastes. Entrepreneurs and BSF facilities will require regulatory approvals and facility upgrades to license these strains and begin turning waste into valuable products. Research about industry and public perceptions will be required to ensure products will be used. Organic waste is an abundant resource worldwide, and worldwide development of this technology may be influenced by access to funding for businesses and infrastructure; government regulatory processes; and whether scientists and BSF facilities can make products that will be adopted by downstream industries. Ultimately, BSF biomanufacturing may reduce landfilling, open dumping and burning of organic wastes and clean up biosolids to safely grow crops and close nutrient cycles.
